# Imaging findings of splenic emergencies: a pictorial review

**DOI:** 10.1007/s13244-016-0467-8

**Published:** 2016-02-16

**Authors:** Emre Unal, Mehmet R. Onur, Erhan Akpinar, Javid Ahmadov, Musturay Karcaaltincaba, Mustafa N. Ozmen, Deniz Akata

**Affiliations:** School of Medicine, Department of Radiology, Hacettepe University, Ankara, Turkey 06100; Department of Radiology, Zonguldak Ataturk State Hospital, Zonguldak, Turkey 67000

**Keywords:** Emergency, Torsion, Trauma, Sequestration, Spleen

## Abstract

**Abstract:**

Although traumatic injuries are the cause of common splenic emergencies in the emergency room, various nontraumatic conditions may also affect the spleen with possible life-threatening results. In this pictorial review, we present imaging findings of usual and unusual splenic emergencies. It is essential to be familiar with key imaging findings and advantages of different modalities to reach a definitive diagnosis.

***Teaching points*:**

• *Delayed splenic rupture is commonly related to subcapsular hematoma*.

• *Subtle haemorrhage is commonly restricted to the site of injury “sentinel clot sign”*.

• *The whorled appearance is the key imaging feature of splenic torsion*.

## Introduction

The spleen is one of the most commonly injured organs in blunt abdominal trauma. Nevertheless, inflammatory and infectious disorders of the spleen are not uncommon. In addition, less common vascular incidents and splenic torsion can also be seen. Ultrasound (US) is usually used for the follow-up of patients with splenic emergencies, since accurate initial diagnosis based solely on US findings is limited. Differentiation of splenic hematoma from abscess or infarct and detection of an active bleeding may not be reliably made by US. Moreover, presence of subcutaneous air bubbles or air bubbles in an abscess cavity may prevent sonographic evaluation.

Therefore, computed tomography (CT) is the main modality used in the assessment of splenic emergencies. CT can demonstrate any subtle parenchymal change related to both traumatic and nontraumatic conditions. Furthermore, acquisition of a CT scan, in arterial phase, in addition to conventional portal venous phase, increases the detection of traumatic contained splenic vascular injuries [1]. In the case of a suspicion, conventional angiography could be considered for detection and also treatment of vascular injury [1]. Nevertheless, conventional angiography is preserved for treatment rather than to diagnose vascular insults, due to increased sensitivity and specificity of multiphasic CT scans in the assessment of splenic vascular emergencies [1, 2].

In clinical practice, the spleen is relatively underrated in the setting of acute abdomen. However, late diagnosed splenic emergencies may result in mortality. The purpose of this paper is to highlight the usual and unusual causes of splenic emergencies with key imaging features.

## Splenic injury

The spleen is a vulnerable and frequently injured visceral organ, particularly in blunt abdominal trauma [3]. Initial radiological examination in a traumatized patient, called focused assessment with sonography for trauma (FAST), is commonly performed by US. Although revealing rib fractures or soft tissue swelling on plain radiographs may raise suspicion for a splenic injury, radiographs have limited use in the emergency room. FAST is performed mainly to detect any free fluid in the abdominal cavity. However, US examination is limited in most cases, for several reasons (subcutaneous emphysema/foreign body, bowel gas and chaotic emergency room). Thus, CT should be the fundamental modality, since it improves the detection of subtle hemoperitoneum with or without visceral injuries and active bleeding.

**Fig. 1:**
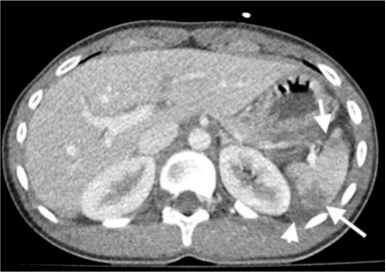
Splenic hematoma. Axial contrast-enhanced CT of a 35-year-old man after a motor vehicle accident (MVA) demonstrates hematomas in splenic parenchyma with low-attenuated appearance (*arrows*). Perisplenic haemorrhage is also noted (*arrowhead*). The splenic capsule is intact

**Fig. 2:**
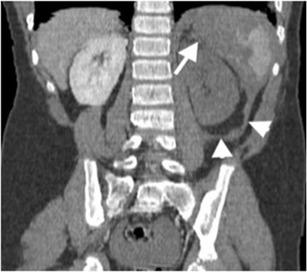
Splenic subcapsular hematoma. Coronal contrast-enhanced CT image of a 18-year-old man demonstrates parenchymal, subcapsular splenic hematoma (*arrow*) and accompanying retroperitoneal hematoma (*arrowheads*). Devascularised left kidney is also noted

CT may demonstrate laceration, hypoperfusion, subcapsular / parenchymal hematoma, active bleeding and pseudoaneurysms in a splenic injury. A parenchymal hematoma is the result of contusion and haemorrhage restricted in parenchyma with intact capsule (Fig. 1) [4]. On the other hand, a subcapsular hematoma is located between the intact capsule and parenchyma with lenticular shape, which is also thought to be responsible for delayed splenic rupture (Fig. 2) [3, 4]. Occasionally, a parenchymal hematoma may not be differentiated from a perfusion alteration caused by vascular insult. However, the triangular shape of the hypodensity that reaches to the splenic hilum may be the clue of a vascular insult and be associated parenchymal change on a contrast-enhanced CT image.

**Fig. 3:**
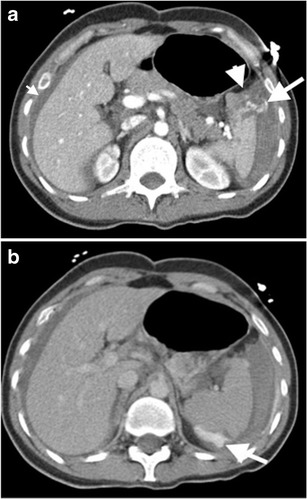
Active bleeding after splenic injury. Axial contrast-enhanced CT images at early (**a**) and delayed (**b**) phase demonstrate splenic laceration (*arrowhead in a*) and active intraperitoneal bleeding (*long arrows in a and b*). Absence of clot formation results from active massive haemorrhage, as clot formation is not fast as bleeding. Extravasated contrast material can accumulate in a dependent part of the body without restriction of any clotted hematoma as of yet (*arrow in b*). Moreover, the presence of perihepatic fluid (*short arrow in a *) supports the evidence of massive bleeding, since blood can act in a pattern similar to the free fluid, due to a decreased percentage of clot formation

Active haemorrhage can be demonstrated by contrast-enhanced CT as an extravasation of intravenously introduced contrast material to the abdominal cavity. However, active extravasation should be evaluated by dual (arterial and venous) phase CT with appropriate contrast injection, since arterial phase images may not demonstrate venous extravasation, and similarly, arterial extravasation may not be differentiated from venous bleeding on venous phase images [3, 4]. Active extravasation can be seen as a focus of linear or nodular hyperdensity within a hematoma or into the abdominal cavity, on arterial phase images [3]. On delayed phase images, accumulation of contrast is seen within or in the dependent portion of hematoma with regard to degree of clot formation (Fig. 3). In the presence of acute massive haemorrhage, hemorrhagic fluid may act in a pattern similar to the free fluid due to decreased percentage of clot formation. In subtle extravasation, haemorrhage is commonly restricted to the site of solid organ injury, with the appearance of what is called the “sentinel clot sign” [3, 4].

## Splenic infarction

**Fig. 4:**
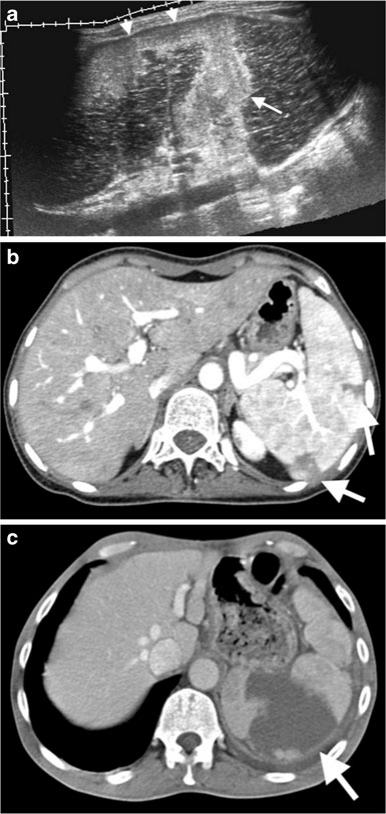
Splenic infarcts with various clinical conditions. (**a**) Panoramic view of gray scale US of a 54-year-old man with atrial fibrillation demonstrates hyperechoic infarct (*arrow*) traversing splenic parenchyma from the hilum to a peripheral part of the spleen. Neighbouring subcapsular portion of the spleen exhibits a hyperechoic appearance (*arrowheads*) representing subcapsular infarct. (**b**) Axial contrast-enhanced CT of a 21-year-old woman with polyarteritis nodosa reveals peripheral wedge-shaped low attenuated infarcts (*arrows*). (**c**) Axial contrast-enhanced CT image of a 79-year-old man with atrial fibrillation reveals a peripheral wedge-shaped low attenuated infarct (*arrow*)

Splenic infarction can be caused by various conditions such as hemoglobinopathies, cardiac emboli, torsion, collagen vascular disease, trauma, splenic artery aneurysm, pancreatic diseases and portal hypertension [2, 3, 5, 6]. Regardless of the aetiology, the main reason is perfusion deficiency, mostly caused by splenic artery injury or occlusion. The appearance of infarction principally depends on the time of the event [3, 5, 6]. In the acute phase of the incident, infarcted splenic parenchyma becomes edematous due to associated inflammation and necrosis, which results in ill-defined areas of decreased attenuation/ echogenicity (Fig. 4). Over time, the region of infarction appears as a sharply demarcated area of volume loss [3, 5]. In the chronic phase, fibrosis or calcification may occur [2, 6].

## Splenic artery aneurysm and pseudoaneurysm

Extensive use of cross-sectional imaging, particularly CT, in clinical practice allowed for the detection of increased numbers of splenic artery aneurysms (SAA) as incidental findings [7, 8]. Although there is a wide range, SAA has an estimated incidence of 0.8 % [7, 8]. Portal hypertension, cirrhosis, liver transplantation, pregnancy, and autoimmune conditions are thought to be responsible for SAA. On the other hand splenic artery pseudoaneurysm may occur due to pancreatitis, trauma, iatrogenic injury, and peptic ulcer disease [2, 7, 8].

**Fig. 5:**
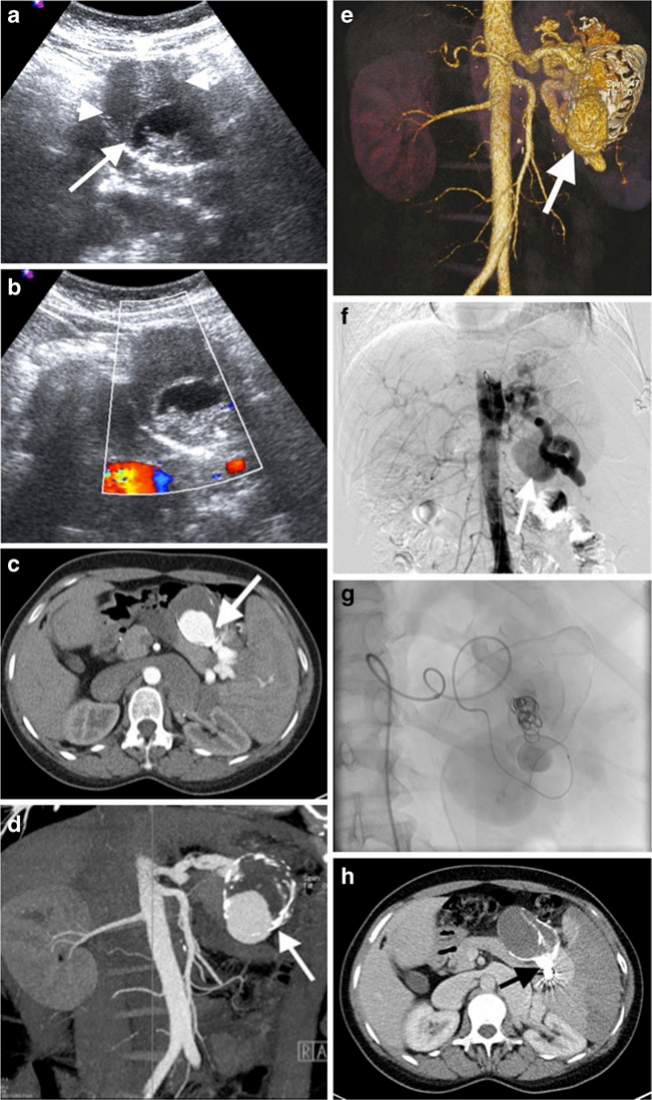
Splenic artery aneurysm. (**a**) Gray scale US of a 45-year-old woman presenting with left upper quadrant pain demonstrates a well-defined mass, with anechoic (*arrow*) and hypoechoic (*arrowheads*) portions representing patent and thrombosed parts of the aneurysm, respectively. (**b**) No flow was detected on colour flow Doppler US. (**c**) Axial contrast-enhanced and (**d**) coronal maximum intensity projection (*MIP*) CT demonstrates splenic artery aneurysm (*arrows*) with partial thrombosis. (**e**) Volume rendering (*VR*) CT image reveals splenic artery aneurysm (*arrow*). (**f**) Digital subtraction angiography (*DSA*) demonstrates aneurysm (*arrow*) arising from splenic artery. (**g**) Coil embolization was performed to embolize the neck of the aneurysm. (**h**) No contrast enhancement was observed (*arrow*) on contrast-enhanced CT after coil embolization

**Fig. 6:**
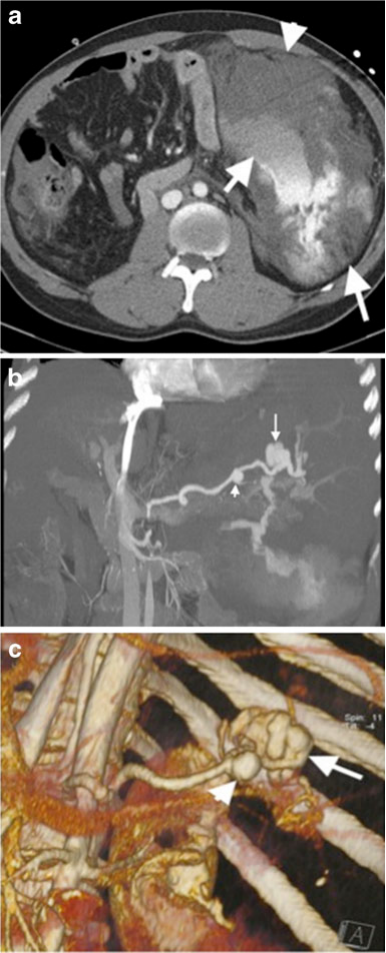
Ruptured splenic artery aneurysm. (**a**) Axial contrast-enhanced CT of a 57-year-old man with shock findings demonstrates a splenic artery aneurysm (*short arrow*) with surrounding active haemorrhage (*arrowhead*). Spleen (*long arrow*) has an heterogeneous appearance secondary to perfusion deficiency resulting from aneurysm haemorrhage. (**b**) Coronal MIP and (**c**) VR CT images reveal a large (*arrows*) and small (*arrowheads*) aneurysms

**Fig. 7:**
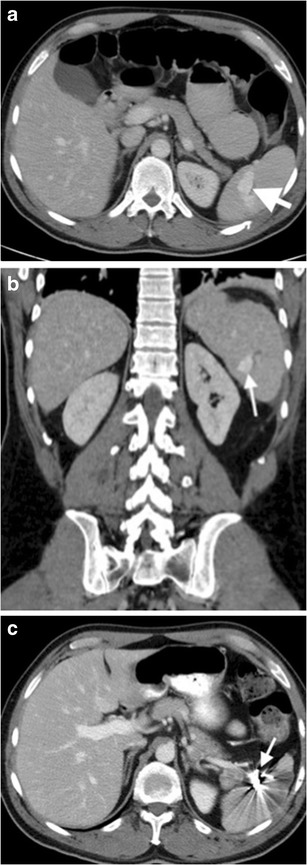
Splenic artery pseudoaneurysm. (**a**) Axial and (**b**) coronal contrast-enhanced CT images of a 45-year-old man with a history of a motor vehicle accident demonstrate a saccular-shaped contrast material filling (*arrows*) in the spleen parenchyma representing pseudoaneurysm of splenic artery. (**c**) Axial contrast-enhanced CT image reveals embolized splenic artery segment (*arrow*) in the splenic hilum

The role of US is limited in the diagnosis of SAA due to technical difficulties. Also, frequently associated mural thrombosis and calcification may prevent detection of colour flow on Doppler US (Fig. 5a and b). SAA are best evaluated on arterial phase CT images (Figs. 5c-h and 6). It has been further reported that reformatted images may also be required to differentiate SAA from small nodular islet cell tumours of the pancreas [7]. Pseudoaneurysm may be distinguished from true aneurysm by imaging with the findings of underlying cause (surrounding inflammation, haemorrhage) and irregular wall (Fig. 7). Moreover, delayed phase images should also be acquired to distinguish active continued bleeding from pseudoaneursym in selected cases, as pseudoaneursym should not grow in size compared to active bleeding, which produces increased size of contrast material accumulation with ill-defined margins (Fig. 3).

It has been advised that although asymptomatic, aneurysms greater than 2 cm should be considered for treatment [7, 8]. Nevertheless, symptomatic aneurysms, regardless of size, pseudoaneurysms, and asymptomatic aneurysms in women of childbearing age or in patients with liver transplantation and portal hypertension are recommended for treatment [7, 8].

## Thrombosis of the splenic artery and vein

**Fig. 8:**
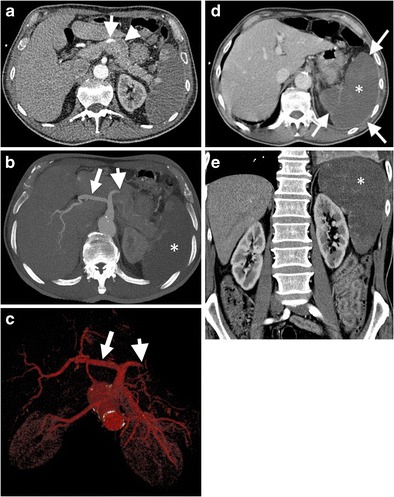
Splenic artery thrombosis. (**a**) Axial contrast-enhanced arterial phase CT of a 74-year-old man with atrial fibrillation demonstrates patent (*arrow*) and thrombosed (*arrowhead*) portions of the splenic artery. (**b**) MIP and (**c**) VR CT images more clearly demonstrate thrombosed splenic (*arrowhead*) and patent hepatic (*arrow*) arteries. (**d**) Axial and (**e**) coronal contrast-enhanced CT images at venous phase reveal diffuse splenic infarct (*asterisks*) resulting from splenic artery thrombosis. Capsular enhancement is seen around the spleen (*arrows in d*)

Splenic vein thrombosis is a relatively common entity in clinical practice, most commonly caused by acute or chronic pancreatitis and pancreatic malignancy [2, 9]. However, splenic artery occlusion is a rare condition that may manifest as entire splenic infarction with only capsule enhancement on CT (Fig. 8) [10]. Capsular enhancement is related to preserved arterial flow from short gastric arteries [2, 10]. Splenic artery is best evaluated on arterial phase CT images (Fig. 8). Infarction can be complicated by abscess formation, haemorrhage, and rupture [2, 3]. However, due to preserved capsular enhancement, rupture is unlikely in splenic infarct. In the case of complication, splenectomy can be considered as a treatment option.

## Splenic torsion

Splenic torsion is a rare entity with an annual incidence of less than 0.2 % [11]. The condition is related to splenic hypermobility (wandering spleen) caused by laxity or absence of the supporting ligaments [12]. In children, the aetiology is thought to be congenital origin with abnormal development of the dorsal mesogastrium. However, in adults, ligamentous laxity is thought to be caused by multiparity, previous splenic surgery/trauma, and splenomegaly [11, 12]. Patients may present with various clinical symptoms, due to intermittent torsion, spontaneous detorsion or splenic infarction [12].

**Fig. 9:**
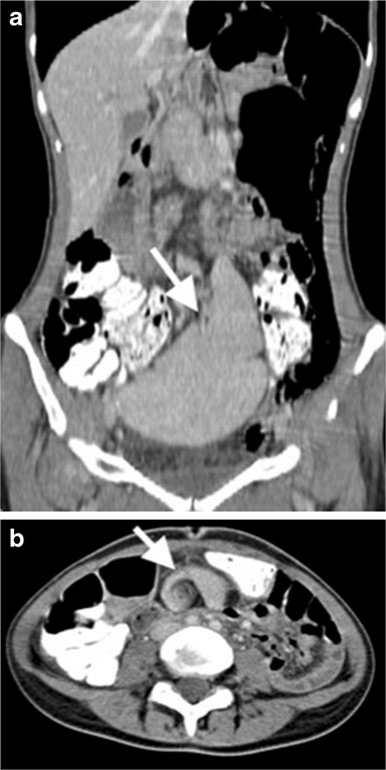
Splenic torsion in a patient with wandering spleen. (**a**) Coronal contrast-enhanced CT image reveals ‘ectopic wandering spleen’ (*arrow*) in the pelvis. (**b**) Axial contrast-enhanced CT at superior level than pelvis further demonstrates *‘whirling’* appearance (*arrow*) of splenic vessels, suggesting *‘torsion of the spleen’* as well. Normal parenchymal enhancement indicates lack of infarction

**Fig. 10:**
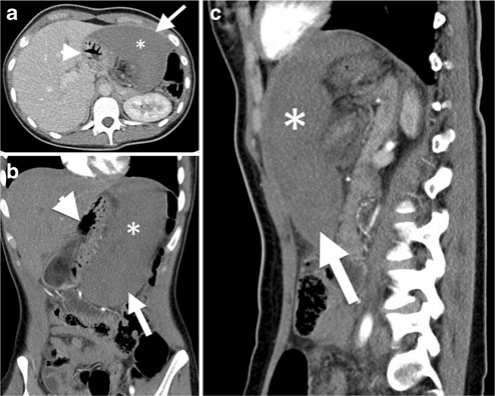
Splenic torsion. (**a**) Axial, (**b**) coronal and (**c**) sagittal contrast-enhanced CT images of a 17-year-old female with a history of recurrent abdominal pain and suspected Familial Mediterranean Fever show anteromedially displaced spleen (*arrows*). No contrast enhancement was observed in spleen parenchyma (*asterisks*), suggesting splenic infarct secondary to splenic torsion. Stomach (*arrowheads*) is posteromedially compressed by enlarged and torsed spleen

The whorled appearance of splenic vascular pedicle on cross-sectional images is a reliable sign of torsion (Fig. 9) [2, 3, 11]. The spleen may be localized in anatomically abnormal localizations. Focal or diffuse infarcted areas may be observed in spleen parenchyma in patients with splenic torsion (Fig. 10). Splenectomy is required in most cases, since patients commonly present with splenic infarction or haemorrhage. Nevertheless, splenopexy can be considered in patients without the findings of splenic infarction (Fig. 9) [12].

## Splenic sequestration

**Fig. 11:**
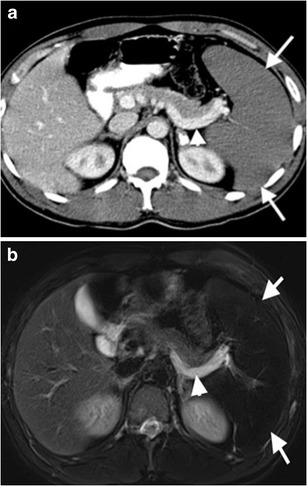
Splenic sequestration. (**a**) CT features of ASSC. Contrast-enhanced axial CT image of a 26-year-old male with known sickle cell-thalassemia reveals lack of enhancement in spleen parenchyma (*arrow*). Retrograde filling of splenic vein (*arrowhead*) from the portal vein can be visualized. (**b**) Axial T2-weighted MRI demonstrates hypointense spleen (*arrows*) secondary to iron deposition. Splenic vein manifests with hyperintense appearance (*arrowheads*) due to slow flow resulting from retrograde venous filling

Acute splenic sequestration crisis (ASSC) is a form of hypovolemic and anaemic crisis, based on massive splenic enlargement due to accumulation and trapment of sickle-shaped cells [13]. Early diagnosis of ASSC is crucial, since rapid pooling of a large volume of blood may result in splenic rupture, hypovolemic shock and death. Enlarged and heterogeneous (due to areas of infarction and haemorrhage) parenchyma with the patency of splenic artery and vein are the key findings for ASSC in sickle cell disease. Blood flow in splenic vein results from retrograde filling of splenic vein from portal vein (Fig. 11).

## Acute splenic infection and abscess

**Fig. 12:**
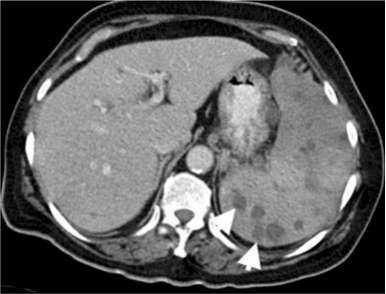
Splenic infection. Axial contrast-enhanced CT of a 35-year-old man with acute myeloblastic leukemia shows low-attenuated multiple focal lesions (*arrowheads*) representing splenic candidiasis

**Fig. 13:**
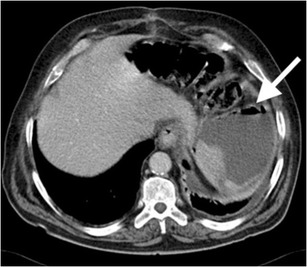
Splenic abscess. Axial contrast-enhanced CT of a 40-year-old woman demonstrates air (*arrow*) within a low attenuated splenic abscess. A culture test was positive for E.coli

Splenic infection may present as a solitary or multiple lesions. The latter is frequently caused by candida albicans, which is a common pathogen in immunocompromised patients (Fig. 12) [2, 9, 14]. Although rare, mycobacterium tuberculosis, histoplasmosis capsulatum and pneumocystis carinii infections may also cause a similar appearance [6, 9, 14, 15]. On the other hand, pyogenic abscess commonly appears as a single lesion. The underlying causes can be listed as follows: hematogenous infection, trauma, infarction, amoebic dysentery, and infective endocarditis [2, 3, 14]. Micro-abscesses are seen as foci of decreased attenuation on both US and CT images (Fig. 12) [2, 3, 6, 14, 15]. The presence of air bubbles and peripheral wall enhancement give the definite diagnosis of pyogenic abscess (Fig. 13).

**Fig. 14:**
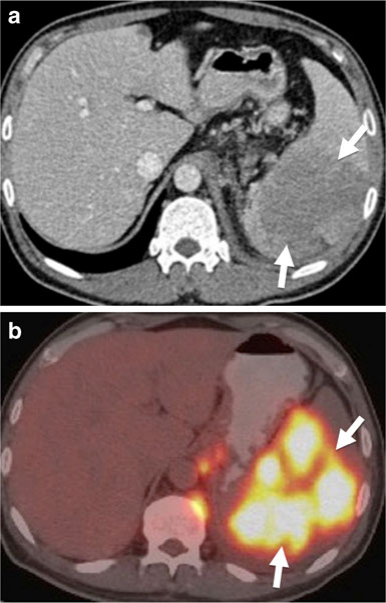
Splenic lymphoma. (**a**) Axial contrast-enhanced CT of a 30-year-old man reveals a large hypodense mass in spleen (*arrows*) (**b**) Increased FDG uptake is also noted (*arrows*)

On the other hand, splenic lymphoma may also appear as multiple nodular lesions, and therefore may mimic splenic infection [16]. Moreover, one should emphasise that splenic lymphoma may rarely present as a single mass [16]. In this case, it may mimic splenic infarction or laceration (Fig. 14). However, increased FDG uptake on PET/CT images suggests the diagnosis of lymphoma (Fig. 14) [14].

## Conclusion

Traumatic injuries comprise an important part of the splenic emergencies. However, the spleen may also be affected by various nontraumatic emergent disorders. Awareness of the imaging findings in splenic emergencies assists in establishing the diagnosis of life-threatening conditions.
